# Opportunity and Threat in Parallel: How Artificial Intelligence Application Predicts Employees’ Proactive Learning Behavior Through Parallel Primary Appraisals

**DOI:** 10.3390/bs16091713

**Published:** 2026-09-21

**Authors:** Yixun Lu, Long Ye, Shaojun Shen

**Affiliations:** 1 School of Economics and Management, Beijing Jiaotong University, Beijing 100044, China; ylu65@dons.usfca.edu; 2 China Transportation Education Research Association, Beijing 100029, China

**Keywords:** artificial intelligence application, proactive learning behavior, opportunity perception, threat perception, proactive personality, cognitive appraisal theory of stress

## Abstract

As organizations embed artificial intelligence (AI) in everyday work, understanding how employees respond has become a pressing question for human resource management. Drawing on the cognitive appraisal theory of stress, this study examines how AI application relates to employees’ proactive learning behavior through two parallel primary appraisals: opportunity perception and threat perception. It also tests proactive personality both as a direct predictor and as a moderator at two theoretically distinct locations: the formation of appraisals and the translation of appraisals into behavior. Using a four-wave panel design among employees from multiple industries, we find that AI application is positively associated with proactive learning behavior overall. This association operates through two opposing pathways: opportunity perception strengthens proactive learning, whereas threat perception suppresses it. Additional analyses indicate that the opportunity pathway is the more robust of the two across longitudinal specifications. The two appraisals are statistically separable and jointly elevated in a subgroup of employees, indicating parallel rather than strictly bipolar appraisal. Proactive personality is positively associated with opportunity perception and proactive learning, negatively associated with threat perception, and moderates the appraisal-formation stage rather than the appraisal-to-behavior stage. These findings extend the cognitive appraisal theory of stress to the AI context and suggest that dispositional proactivity operates mainly by shaping how employees read AI, rather than by changing how they act on an appraisal once it has formed.

## 1. Introduction

Artificial intelligence (AI) is reshaping how organizations operate. The “AI+” initiative in China’s 15th Five-Year Plan calls for AI integration across economic sectors, and firms worldwide are embedding intelligent systems into everyday workflows to raise performance and innovation (Tang et al., 2022). Returns on these investments remain uneven: some employees treat AI as a developmental resource and upgrade their skills, while others read it as a threat to job security and disengage from learning (Brougham & Haar, 2018; Tu et al., 2023). Employees are the “last mile” through which AI creates organizational value (Zou et al., 2023), and their willingness to update knowledge and skills conditions whether that value is realized. Deloitte’s (2026) Global Human Capital Trends report underscores the gap: while 85% of business leaders regard adaptive capacity as vital, only 7% say their organizations are leading in this area.

Despite growing interest in how AI transforms work, research has concentrated on distal outcomes such as task performance, creative behavior, and job displacement (Long et al., 2020; Sheng et al., 2022; M. Li et al., 2026), and on employees’ intentions to support digital-intelligence transformation more broadly (Wu et al., 2025a). How AI application relates to proactive learning behavior (self-initiated, anticipatory effort to update skills and knowledge; Zou et al., 2023) remains less well understood.

Proactive learning is a demanding outcome to explain precisely because it is self-initiated. By definition it is not elicited on cue; individuals differ in how much of it they do irrespective of any particular workplace event. Our claim is therefore not that AI application “triggers” learning in a reactive sense, but that it alters the interpretive context within which employees decide how much anticipatory effort to invest. Cognitive appraisal theory specifies this mechanism: a salient change in the work environment is evaluated for its implications for personal well-being, and that evaluation shapes which coping strategies, including deliberate skill-building, appear worthwhile (Lazarus & Folkman, 1984). On this account proactive learning remains forward-looking and self-initiated; what varies with appraisal is its intensity. Consistent with this reading, we control for employees’ baseline level of proactive learning throughout, so that our estimates speak to variation around each person’s own established tendency rather than to its origin.

Studies of double-edged technology effects have relied chiefly on two frameworks. The challenge–hindrance stressor framework (Cavanaugh et al., 2000; LePine et al., 2005) traces positive and negative effects to different, objectively distinguishable stressors. The approach–avoidance motivation framework and regulatory focus theory (Higgins, 1997; Yan et al., 2025) treat promotion and prevention orientations as poles of a single motivational continuum. Neither framework is incapable in principle of accommodating mixed responses: a single event can be decomposed into challenge and hindrance components, and regulatory focus can be measured as two separable scales. Our argument is narrower: these frameworks locate the duality either in the stressor or in a chronic orientation, whereas cognitive appraisal theory locates it in the appraisal process itself, and therefore predicts that the same person may hold two opposed evaluations of the same technology at the same time. That prediction is directly testable, and testing it is one purpose of this study.

We accordingly root our model in the cognitive appraisal theory of stress (Lazarus & Folkman, 1984) and propose that AI application activates both opportunity perception and threat perception, that the two appraisals are relatively independent rather than bipolar, and that they transmit effects through parallel, oppositely signed paths. We test this parallel-appraisal claim directly rather than inferring it from a near-zero correlation alone, while noting at the outset that a panel design of this kind can establish separability and joint elevation but cannot demonstrate strict within-person simultaneity.

We further introduce proactive personality, and specify its role more completely than prior work has done. We estimate its direct associations with both appraisals and with proactive learning, and we test its moderating role at two locations simultaneously: the stage at which AI application is translated into primary appraisals, and the stage at which appraisals are translated into learning. Studies testing a single location cannot determine whether dispositional traits shape how employees read a stressor or how they act on their reading. Because the first stage is logically prior, we develop and report it first.

A final contribution concerns method. Most AI-outcome studies use cross-sectional or two-wave designs, which makes temporal precedence across the antecedent–mediator–outcome chain difficult to establish (Cole & Maxwell, 2003). We use a four-wave panel, place each construct at a distinct time point, verify longitudinal measurement invariance, and, departing from common practice, include baseline levels of the mediators and of the outcome in the primary hypothesis tests rather than only in supplementary robustness checks. Because baseline adjustment materially changes what is supported, we report the adjusted and unadjusted estimates side by side and interpret the difference substantively rather than treating it as a nuisance. Throughout we describe our results as longitudinal prediction and temporal association; the design improves temporal ordering but does not identify causal effects.

## 2. Theory and Hypotheses

### 2.1. Cognitive Appraisal Theory of Stress

The cognitive appraisal theory of stress (Lazarus & Folkman, 1984) holds that individuals continuously evaluate environmental events for their relevance to personal well-being (primary appraisal) and for their own capacity to cope (secondary appraisal). Primary appraisal classifies events as threatening (potential harm or loss) or as challenging and opportunity-laden (potential mastery or gain); secondary appraisal assesses available coping resources (Jiang & Wang, 2022). The two forms of appraisal unfold dynamically rather than in fixed sequence.

Appraisal outcomes channel coping. When a stressor is appraised as threatening and controllability is judged low, individuals tend toward emotion-focused coping such as avoidance, distancing, withdrawal (Hobfoll, 1989; Lazarus & Folkman, 1984). When a stressor is appraised as challenging and potentially controllable, individuals tend toward problem-focused coping such as skill-building, help-seeking, behavioral adaptation (Frese & Fay, 2001; Parker et al., 2006). The property that matters most for our purposes is that the theory permits two primary appraisals to be held simultaneously about the same stressor, which makes it well suited to theorizing how one technology can produce heterogeneous responses within the same person.

### 2.2. AI Application and Proactive Learning Behavior

AI application refers to the degree to which employees invest time and effort using AI technologies, including machine vision, intelligent robotics, and natural language processing, to accomplish work tasks (Tang et al., 2022; W. Wang & Liu, 2025). Proactive learning behavior is a form of proactive employee behavior: self-initiated, anticipatory effort to acquire knowledge and skills in order to improve oneself and one’s work context (Crant, 2000; Zou et al., 2023). Within a career-adaptability lens, it is both an adaptability resource and an active response to technological uncertainty (Fugate et al., 2004; Parker et al., 2010).

Proactive learning is especially important in the AI context because the value of AI implementation depends not only on the technology itself but also on employees’ willingness to update the knowledge and skills needed to work with it. Although proactive learning is self-initiated rather than mechanically triggered by external events, workplace changes can alter the conditions under which employees choose to invest such effort. AI application changes task requirements, skill expectations, and employees’ perceptions of future work demands. It may therefore shape the extent to which employees express proactive learning tendencies in a given period.

According to the cognitive appraisal theory of stress, employees evaluate work changes in terms of their implications for personal well-being and future goals. When AI is appraised as an opportunity, employees are more likely to see learning as a useful problem-focused coping strategy that allows them to master the technology and benefit from its affordances (Tang et al., 2022; W. Wang & Liu, 2025). Although AI may also evoke threat appraisals, the overall association between AI application and proactive learning is expected to be positive because AI use often makes new skill demands salient.

**Hypothesis** **1.** *AI application positively predicts employees’ proactive learning behavior.*

### 2.3. The Parallel Mediating Roles of Opportunity and Threat Perception

AI application can elicit two forms of primary appraisal (Brougham & Haar, 2018; M. Li et al., 2026): opportunity perception, which frames AI as a source of knowledge expansion and efficiency gain, and threat perception, which frames AI as a source of skill devaluation and displacement risk. Following the cognitive appraisal theory of stress, we treat these as separate evaluative dimensions rather than poles of one continuum (Highhouse & Yüce, 1996; Lazarus & Folkman, 1984), and therefore as capable of being held in parallel by the same individual.

**The opportunity pathway.** When employees construe AI as an opportunity, they are drawn toward problem-focused, adaptive coping (Lazarus & Folkman, 1984). AI assistance lowers cognitive load on routine and high-risk tasks and surfaces information more efficiently (Tang et al., 2022), supporting positive affect, task engagement, and creative self-efficacy (M. Li et al., 2026; Wu et al., 2025b). In this state employees are motivated to update knowledge structures and acquire the skills needed to work alongside AI.

**The threat pathway.** When employees construe AI as a threat, anxiety and defensive motivation are activated (Highhouse & Yüce, 1996; Tarafdar et al., 2007). Perceived risk of skill obsolescence and replacement gives rise to technology-induced job insecurity (Ashford et al., 1989; Tu et al., 2023). Where controllability perceptions are limited and retraining resources scarce, threat appraisal more commonly triggers emotion-focused coping such as withdrawal, avoidance, reduced exploratory investment (Hobfoll, 1989; Y. Wang & Zhu, 2026), thereby suppressing proactive learning.

Although the appraisal–learning relationship could in principle be nonlinear, we expect the focal associations to be predominantly monotonic in the present context. For threat perception, a modest sense of risk might motivate some compensatory learning when employees believe the situation remains controllable. However, the AI-related threats emphasized in this study concern skill devaluation, job insecurity, and professional-status loss. As such appraisals intensify, they are more likely to signal low control and to elicit anxiety, defensive coping, and withdrawal from exploratory learning rather than sustained skill investment. For opportunity perception, very high opportunity appraisal could theoretically produce diminishing returns if employees already perceive sufficient learning benefits. Nevertheless, the central meaning of opportunity appraisal is that learning is useful, controllable, and instrumentally valuable for adaptation. We therefore expect stronger opportunity perception to be associated with greater proactive learning, and stronger threat perception to be associated with lower proactive learning. Together the two paths constitute a dual mechanism in which AI application is simultaneously associated with higher proactive learning through opportunity perception and lower proactive learning through threat perception.

**Hypothesis** **2a.** *Opportunity perception mediates the relationship between AI application and proactive learning behavior, such that the indirect association is positive.*

**Hypothesis** **2b.** *Threat perception mediates the relationship between AI application and proactive learning behavior, such that the indirect association is negative.*

**Hypothesis** **3a.** *Opportunity perception positively predicts proactive learning behavior.*

**Hypothesis** **3b.** *Threat perception negatively predicts proactive learning behavior.*

### 2.4. Proactive Personality: Direct Associations and Location-Dependent Moderation

Proactive personality is a stable dispositional tendency to identify opportunities and act to change one’s circumstances, and a key driver of proactive motivation and behavior (Bateman & Crant, 1993; Crant, 2000; Parker et al., 2010). Prior treatments have generally introduced it as a moderator without specifying its direct associations, which leaves the moderation estimates difficult to interpret. We therefore specify its direct associations explicitly (Hypotheses 6a–6c), in addition to its two candidate moderating roles.

**First-stage moderation (Hypotheses 4a/4b).** The cognitive appraisal theory of stress places stable dispositions among the antecedents of primary appraisal (Lazarus & Folkman, 1984). Because appraisal formation is logically prior to appraisal enactment, we consider this location first. Employees high in proactive personality should convert a given level of AI application into opportunity appraisal more readily, while their higher self-efficacy and sense of occupational control should attenuate catastrophic interpretations of skill displacement (Fugate et al., 2004; Tu et al., 2023). Employees low in proactive personality are more likely to absorb the threatening valence of AI passively.

**Hypothesis** **4a.** *Proactive personality moderates the first-stage relationship between AI application and opportunity perception, such that the positive indirect association via opportunity perception is stronger at higher levels of proactive personality.*

**Hypothesis** **4b.** *Proactive personality moderates the first-stage relationship between AI application and threat perception, such that the negative indirect association via threat perception is weaker at higher levels of proactive personality.*

**Second-stage moderation (Hypotheses 5a/5b).** Alternatively, proactive personality may operate on the behavioral translation of an appraisal that has already formed. Proactive employees are more willing to invest effort under uncertainty and more likely to act on their appraisals (Frese & Fay, 2001; Parker et al., 2006), which could strengthen the opportunity–learning path; their control orientation might also buffer the suppressive effect of threat by reframing it as challenge (Bateman & Crant, 1993; Parker et al., 2010).

**Hypothesis** **5a.** *Proactive personality moderates the second-stage relationship between opportunity perception and proactive learning behavior, such that the positive indirect association is stronger at higher levels of proactive personality*.

**Hypothesis** **5b.** *Proactive personality moderates the second-stage relationship between threat perception and proactive learning behavior, such that the negative indirect association is weaker at higher levels of proactive personality.*

We emphasize that Hypotheses 4 and 5 are stated at the level of the indirect effect and are evaluated with the index of moderated mediation (Hayes, 2015). Moderation of an individual path is a necessary but not sufficient condition for moderated mediation, and we report the two separately throughout so that the distinction remains visible.

**Direct associations.** Employees high in proactive personality habitually scan the environment for opportunities they can exploit and situations they can control (Crant, 2000; Grant & Ashford, 2008), which should raise opportunity appraisal and lower threat appraisal. Because proactive individuals also invest more effort in self-development independent of any particular stressor, proactive personality should predict proactive learning directly.

**Hypothesis** **6a.** *Proactive personality is positively associated with opportunity perception.*

**Hypothesis** **6b.** *Proactive personality is negatively associated with threat perception.*

**Hypothesis** **6c.** *Proactive personality is positively associated with proactive learning behavior.*

**Figure 1:**
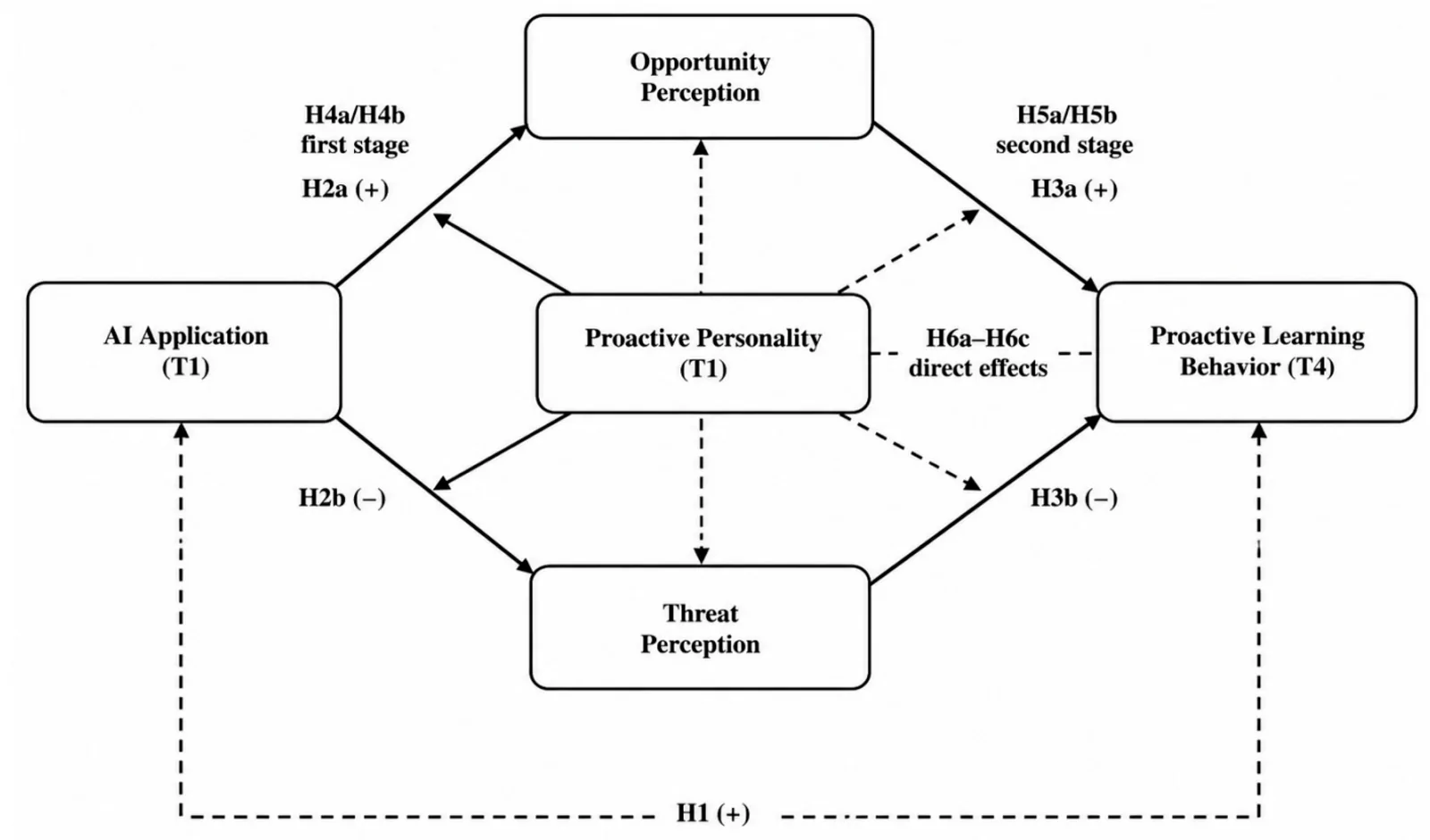
The moderated dual-mediation model. *Note.* Solid arrows denote hypothesized mediating paths; the routed dashed arrow denotes the direct association; arrows terminating on a path denote moderation; dotted arrows denote the hypothesized direct associations of proactive personality. Baseline levels of both mediators and of the outcome are controlled in all estimated models but omitted here for legibility.

The full model, including the direct associations and the two competing moderation locations, is depicted in Figure 1.

## 3. Method

### 3.1. Sample and Procedure

We recruited employees from organizations in which AI technologies are actively used in daily work tasks, spanning manufacturing, services, construction, high technology, public institutions, government agencies, and educational settings. Ethical approval was obtained from the Science and Technology Ethics Committee of Beijing Jiaotong University prior to data collection; all participants provided informed consent, were assured of voluntary participation, and were told their responses would be used only in aggregated, anonymous form.

We employed a four-wave design with approximately one-month intervals. Participants set their own anonymous matching codes to enable cross-wave linking while preserving confidentiality. Wave 1 (T1; March 2025) measured AI application, proactive personality, and baseline values of both mediators. Wave 2 (T2; April 2025) measured baseline proactive learning behavior. Wave 3 (T3; May 2025) measured opportunity and threat perception. Wave 4 (T4; June 2025) measured proactive learning behavior. Baseline mediators and the baseline outcome enter the primary hypothesis tests, not merely supplementary checks.

Two intervals require justification. The first is the antecedent–mediator interval of two months (T1 → T3). Appraisals of a durable technological change are not formed instantaneously; they consolidate as employees accumulate experience with the system, and a two-month window allows this while limiting attrition and contamination by unrelated events (Cole & Maxwell, 2003). The second is the mediator–outcome interval of one month (T3 → T4). Proactive learning can in principle be initiated at any time, so this interval should be read not as the time required for the behavior to appear but as the observation window within which its intensity is assessed. We note explicitly that we do not assume one interval is optimal for both appraisal processes. Prior evidence and our own results suggest that threat appraisal reflects a longer-horizon evaluation of occupational risk than opportunity appraisal does (Tu et al., 2023), and we therefore report in Section 4.8 a supplementary analysis using a three-month appraisal window (T1 appraisals → T4 outcome) alongside the one-month window. The lag is therefore treated as a substantive parameter rather than as a design constant.

**Figure 2:**
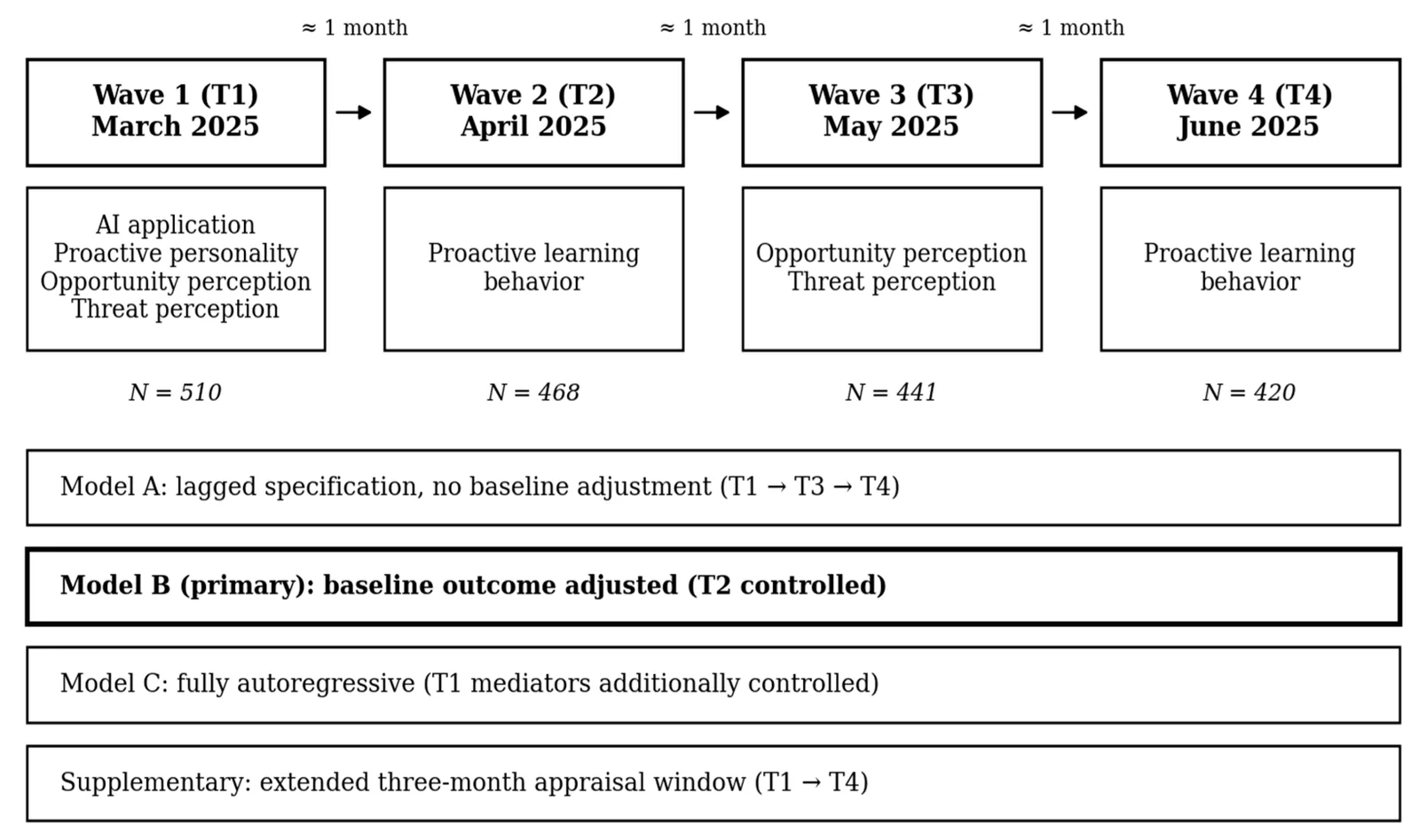
Four-wave design, wave-by-wave sample sizes, and the three analytic models. *Note.* Model A is the unadjusted lagged specification, reported for comparability only. Model B is the primary specification and adjusts for the baseline outcome (T2). Model C is fully autoregressive and additionally adjusts for the baseline mediators (T1). The supplementary analysis extends the appraisal window to three months. The bold type and heavier border of the Model B box identify the primary specification; *N* = number of participants retained at each wave.

Questionnaires were administered anonymously with instructional manipulation checks embedded at Waves 3 and 4, supplemented by response-time filters and straight-lining screening. The design is depicted in Figure 2.

Of 510 responses collected across four waves, 90 were excluded: 15 for failing the Wave 3 attention check, 15 for failing the Wave 4 attention check, 15 for straight-lining, 15 for excessively short response times, 15 for missing Wave 3 match codes, and 15 for missing Wave 4 match codes. The final matched analytic sample comprised 420 participants (82.4% of those initially recruited).

To test for systematic attrition, we compared retained (*N* = 420) and excluded (*N* = 90) participants on all Wave 1 self-report variables. No significant differences emerged on AI application (*t* = 0.03, *p* = 0.977), opportunity perception (*t* = −1.38, *p* = 0.167), threat perception (*t* = −0.49, *p* = 0.624), or proactive personality (*t* = 0.71, *p* = 0.476), indicating that attrition was essentially random with respect to the focal constructs.

A sensitivity power analysis indicated that *N* = 420, with α = 0.05 (two-tailed), nine predictors in the largest regression model, and 80% target power, allows detection of effects as small as *f*^2^ = 0.019, below the smallest effect sizes obtained.

The sample was 46.9% male and 53.1% female. Age: 22.9% aged ≤ 25 years, 59.0% aged 26–45, 18.1% aged ≥ 46. Education: 57.6% held a bachelor’s degree or above (14.0% postgraduate). Tenure: 82.9% had ≥3 years’ experience. Hierarchical level: 53.1% frontline employees, 46.9% managers. Industry: services (24.8%), manufacturing (23.3%), public institutions (14.3%), high technology (10.2%), construction (8.8%), education (8.6%), government (5.7%), and other (4.3%).

### 3.2. Measures

All constructs were measured with validated scales adapted to the AI application context; English-language scales were translated into Chinese following a translation–back-translation procedure (Brislin, 1980), with discrepancies resolved by two bilingual organizational-behavior scholars. Unless otherwise noted, items used a 5-point Likert scale (1 = *strongly disagree*, 5 = *strongly agree*). Reverse-scored items were recoded as (6 − original score). The complete item set is reproduced in Appendix A so that readers can evaluate content validity directly.

**AI application (3 items; T1).** Adapted from Tang et al. (2022) and W. Wang and Liu (2025). A representative item is “Most of my work involves using artificial intelligence technology.” (α = 0.848).

**Opportunity perception (4 items, 1 reverse-scored; T1 and T3).** Adapted from Brougham and Haar (2018) and M. Li et al. (2026). A representative item is “Using AI technology offers many benefits.” (T3 α = 0.872; T1 α = 0.859).

**Threat perception (7 items, 3 reverse-scored; T1 and T3).** Adapted from Brougham and Haar (2018) and M. Li et al. (2026). A representative item is “I worry that AI will one day replace my job.” (T3 α = 0.910; T1 α = 0.913).

**Proactive personality (4 items; T1).** The four-item version of Bateman and Crant’s (1993) scale (α = 0.841).

**Proactive learning behavior (8 items; T2 and T4).** Adapted from Zou et al. (2023). A representative item is “I continually work to improve my work-related skills.” (T4 α = 0.927; T2 α = 0.926).

**Control variables.** We controlled for gender, age, education, organizational tenure, and hierarchical level, each associated with proactive and adaptive behavior in prior research (Crant, 2000; Parker et al., 2006). Robustness analyses using dummy-coded ordinal controls produced identical inferences for all focal paths.

### 3.3. Analytic Strategy

Step 1: confirmatory factor analysis (CFA) of the five-factor measurement model with nested alternatives. Step 2: longitudinal measurement invariance for the repeatedly measured constructs, using configural, metric, and scalar models; invariance is supported when ΔCFI ≤ 0.010 and ΔRMSEA ≤ 0.015 (Cheung & Rensvold, 2002). Step 3: diagnostic screening for common method variance (Podsakoff et al., 2003). Step 4: hierarchical OLS regression with bias-corrected bootstrap confidence intervals (5000 draws) for the parallel mediation tests (Hayes, 2018). Step 5: moderated mediation at the first stage and then the second stage, with the index of moderated mediation as the inferential criterion (Edwards & Lambert, 2007; Hayes, 2015; Preacher et al., 2007). Variables entering interaction terms were standardized beforehand; variance inflation factors remained below 4.2 throughout, and below 2.0 in the primary specification.

Specification of baseline adjustment. This choice determines what our tests mean and is therefore stated explicitly. Three specifications are reported side by side in Section 4.4. Model A is the lagged model without baseline adjustment, included only for comparability with the existing literature. Model B, our primary specification, adds the baseline outcome (T2 proactive learning) to every equation; the T4 outcome is therefore adjusted for its own baseline, and because T2 precedes both the T3 mediators and the T4 outcome, including it in the mediator equations is temporally admissible and makes the effect decomposition exact. Model C is fully autoregressive, adding baseline mediators (T1 opportunity and threat perception) so that the T3 mediators enter as residualized change. Model C is the most stringent specification available in these data, and it is reported as a primary result rather than as supplementary material because it materially qualifies one of our hypotheses. Section 4.8 adds an extended-window analysis in which appraisals measured at T1 predict the T4 outcome adjusted for its T2 baseline.

## 4. Results

### 4.1. Confirmatory Factor Analysis and Discriminant Validity

We estimated five CFA models via maximum likelihood (Table 1). The hypothesized five-factor model fit well (χ^2^ = 304.83, *df* = 289, χ^2^/*df* = 1.055, RMSEA = 0.011, CFI = 0.998, TLI = 0.997, SRMR = 0.030) and better than all nested alternatives. Standardized loadings ranged from 0.619 to 0.751 (all *p* < 0.001). Composite reliability ranged from 0.841 to 0.927 and average variance extracted from 0.569 to 0.651 (Table 2). The square root of each AVE (0.754–0.807) exceeded all inter-construct correlations (maximum |*r*| = 0.678), satisfying the Fornell–Larcker criterion.

### 4.2. Longitudinal Measurement Invariance

Because opportunity perception, threat perception, and proactive learning behavior were each measured at two waves, we verified that loadings and intercepts were equivalent across time before drawing inferences involving change (Cole & Maxwell, 2003). Residual correlations were specified between same-item pairs across waves. Results appear in Table 3.

**Conclusion.** For all three constructs, constraining loadings and then intercepts to equality produced negligible decrements in fit (all |ΔCFI| ≤ 0.001, all |ΔRMSEA| ≤ 0.007), well within the Cheung and Rensvold (2002) criteria. Scalar invariance is therefore supported, which licenses the cross-wave comparisons and the autoregressive specifications reported below: observed differences between waves can be attributed to change in the constructs rather than to change in how the items function.

### 4.3. Descriptive Statistics, Temporal Stability, and Common Method Variance

Means, standard deviations, and intercorrelations for all study variables, including baseline measures, are reported in Table 4. AI application correlated positively with opportunity perception (*r* = 0.468, *p* < 0.001) and threat perception (*r* = 0.425, *p* < 0.001). Opportunity perception correlated positively with proactive learning (*r* = 0.678, *p* < 0.001) and threat perception negatively (*r* = −0.247, *p* < 0.001). Proactive personality correlated positively with proactive learning (*r* = 0.459, *p* < 0.001) and opportunity perception (*r* = 0.328, *p* < 0.001) and negatively with threat perception (*r* = −0.223, *p* < 0.001). Opportunity and threat perception were essentially uncorrelated at T3 (*r* = 0.007, n.s.); the same held at T1 (*r* = −0.043, n.s.).

**Temporal stability.** Cross-wave correlations were 0.823 for opportunity perception (T1–T3), 0.858 for threat perception (T1–T3), and 0.533 for proactive learning behavior (T2–T4). Both appraisals are thus highly stable over one month, while the outcome is moderately stable. This asymmetry is important for interpreting the autoregressive results: when a predictor is stable at *r* ≈ 0.86, very little residual variance remains for a one-month lagged antecedent to explain, and a null residual-change coefficient is not equivalent to the absence of a relationship.

**Education and hierarchical level.** Education correlated negatively with hierarchical level (*r* = −0.122, *p* = 0.012). The direction is correct as reported and is not a coding error. In this sample, the more highly educated respondents were disproportionately concentrated in professional and technical frontline roles, particularly in public institutions, education, and high technology, whereas supervisory positions in manufacturing, construction, and services were more often held by respondents with vocational or secondary qualifications and longer tenure. The association is small and is retained as a control.

We addressed common method variance through procedural controls (temporal separation across four waves, anonymity, mixed item valence) and statistical screening (Podsakoff et al., 2003). Harman’s single-factor test extracted four factors with eigenvalues >1, the first explaining 32.27% of variance; the single-factor CFA fit poorly (RMSEA = 0.154, CFI = 0.531). We regard these as diagnostic checks rather than evidence that common method variance is absent. Neither procedure can establish the absence of method bias, and because all focal variables are employee self-reports, some inflation of the observed associations remains possible. This qualification applies to the main results and not only to the limitations discussed in Section 5.3; supervisor ratings and objective AI-use records would be required to resolve it.

### 4.4. Baseline-Adjusted Mediation Tests (Hypotheses 1, 2a, 2b, 3a, 3b)

**Figure 3:**
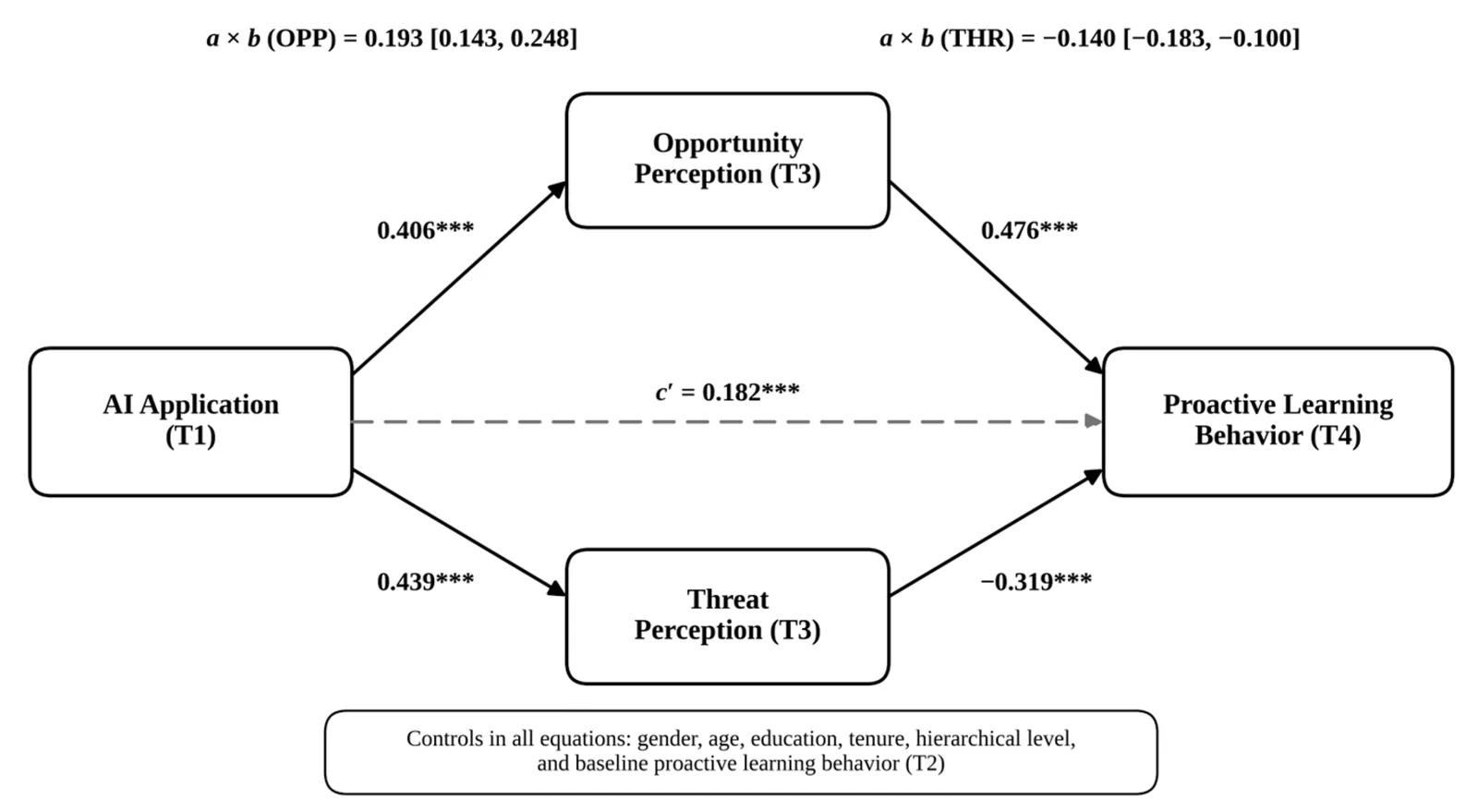
Baseline-adjusted parallel mediation (primary specification). *Note.* Standardized coefficients. *a* × *b* = indirect association, with its 95% bias-corrected bootstrap confidence interval in brackets; *c*′ = direct association. All equations control gender, age, education, tenure, hierarchical level, and baseline proactive learning behavior (T2). *** *p* < 0.001.

Hierarchical regression results are reported in Table 5 for all three specifications, and bootstrap indirect effects in Table 6. Figure 3 presents the primary (Model B) path diagram.

**Primary specification (Model B).** Adjusting for the baseline outcome, AI application predicted opportunity perception (β = 0.406, *p* < 0.001) and threat perception (β = 0.439, *p* < 0.001). With both mediators entered, opportunity perception positively (β = 0.476, *p* < 0.001) and threat perception negatively (β = −0.319, *p* < 0.001) predicted proactive learning, supporting H3a and H3b. The total association was positive (*c* = 0.235, 95% CI [0.136, 0.333]; H1 supported), as was the direct association (*c*′ = 0.182, 95% CI [0.106, 0.258]). The indirect association via opportunity perception was positive (0.193, 95% CI [0.143, 0.248]; H2a supported) and via threat perception negative (−0.140, 95% CI [−0.183, −0.100]; H2b supported). Because the baseline outcome enters every equation, the decomposition is exact: 0.193 − 0.140 + 0.182 = 0.235.

**Comparison with the unadjusted model (Model A).** Without baseline adjustment the same paths were larger (0.276 and −0.141, total *c* = 0.328). Baseline adjustment therefore reduces the opportunity path by roughly 30% while leaving the threat path essentially unchanged, which is what one would expect if part of the unadjusted opportunity association reflects stable between-person differences in learning propensity.

**Fully autoregressive specification (Model C).** When baseline mediators are added so that the T3 appraisals enter as residualized change, the results diverge sharply. The indirect association via opportunity perception remained significant, though much smaller (0.046, 95% CI [0.020, 0.077]). The indirect association via threat perception was not significant (−0.003, 95% CI [−0.013, 0.004]), because AI application no longer predicted change in threat perception (β = 0.031, *p* = 0.356) and the mediator–outcome path itself weakened to marginal significance (β = −0.103, *p* = 0.077).

**Interpretation.** The longitudinal evidence for the threat pathway is substantially weaker than for the opportunity pathway, and H2b must be qualified accordingly: it is supported when the outcome is adjusted for its baseline, and not supported when the mediator is additionally residualized on its own baseline. Substantively, this means AI application is associated with the level of threat appraisal employees hold, not with month-to-month revision of that appraisal. Given a one-month stability of *r* = 0.858, the design has limited power to detect such revision even were it present; the null is therefore suggestive about timescale but not decisive about mechanism. Section 4.8 pursues this directly.

### 4.5. Separability and Joint Elevation of the Two Primary Appraisals

**Figure 4:**
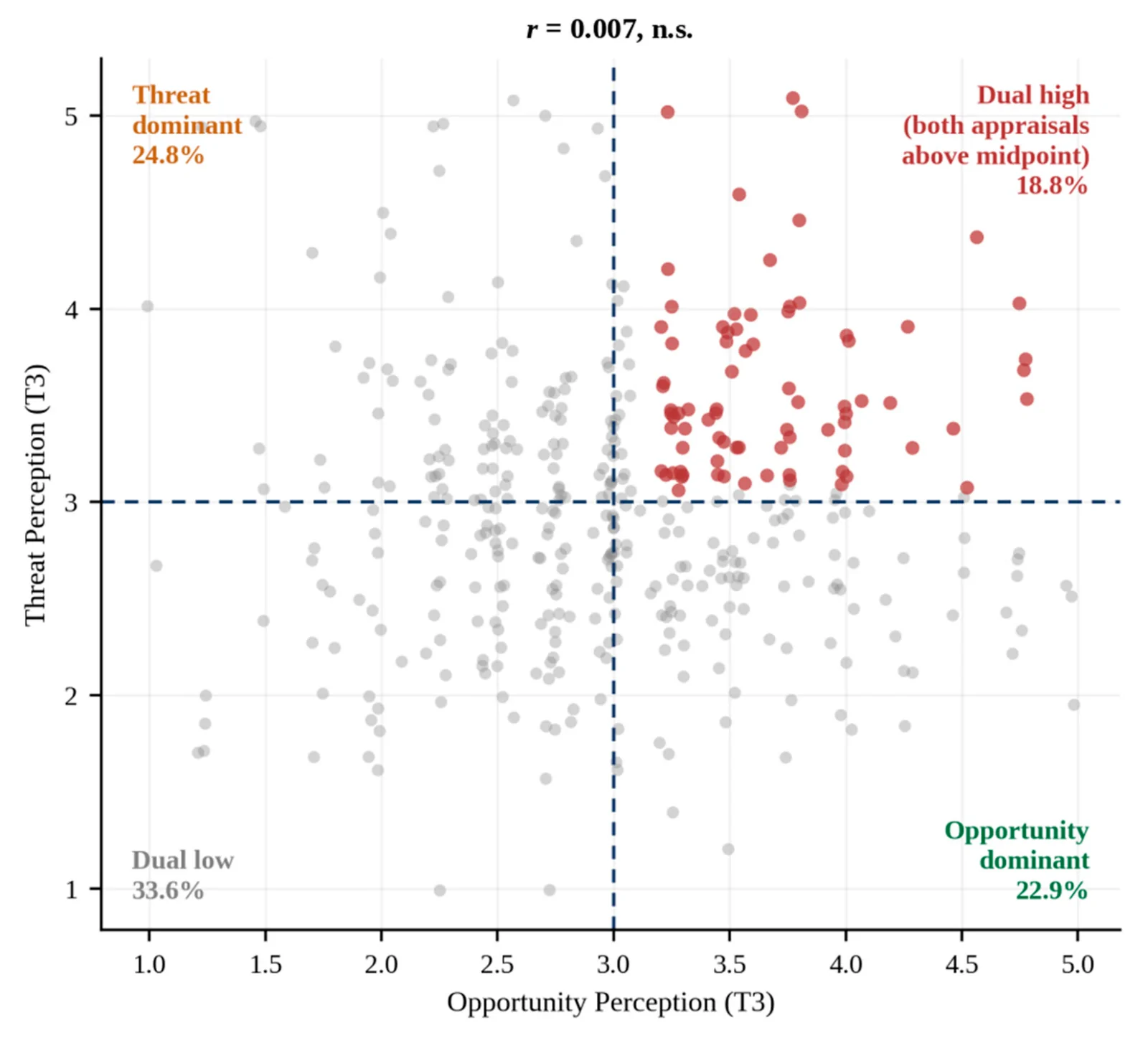
Joint distribution of opportunity and threat perception at T3. *Note*. Reference lines mark the scale midpoint (3.0). Dark red points are respondents scoring above the midpoint on both appraisals (18.8% of the sample). Grey points are all other respondents; the quadrant labels report the percentage of respondents in each quadrant, and their colors serve only to identify the quadrants. *r* = Pearson correlation; n.s. = not significant.

The claim that the two appraisals operate in parallel requires more than a near-zero correlation. A near-zero between-person correlation establishes statistical independence at the sample level; it does not by itself demonstrate that individuals hold both appraisals simultaneously. In principle the same correlation could arise if each person held exactly one appraisal and the two types were equally prevalent. We therefore examined the joint distribution directly (Figure 4).

Using the scale midpoint (3.0) as the reference, 18.8% of respondents scored above the midpoint on both opportunity and threat perception, 22.9% were opportunity-dominant, 24.8% threat-dominant, and 33.6% scored below the midpoint on both. A median-split cross-tabulation showed no association between the two classifications, χ^2^(1) = 0.20, *p* = 0.653. The independence replicated at T1 (*r* = −0.043, n.s.), and change scores between T1 and T3 were also unrelated (*r* = 0.033, *p* = 0.500), indicating that the two appraisals do not merely fail to correlate in level but also move independently over time.

This evidence is consistent with parallel appraisal and inconsistent with strict bipolarity, which would require the dual-high cell to be sparse. It falls short, however, of the within-person evidence that would be ideal. Nearly one in five employees holding both appraisals above the midpoint is a substantial minority, not a majority, and our design cannot establish that the two appraisals are active simultaneously within a single moment of experience rather than alternating within a period. We therefore use the term parallel appraisals throughout, rather than claiming that simultaneous coexistence has been demonstrated, and frame the finding as evidence that opportunity and threat appraisals are separable and jointly held by a substantial subgroup, and identify experience-sampling designs as the appropriate next step. We consider an alternative interpretation of the null correlation in Section 5.1.

### 4.6. Moderated Mediation at the First Stage (Hypotheses 4a/4b and 6a/6b)

Following the logical ordering of the model, we test first-stage moderation before second-stage moderation. Regression results appear in Table 7 and conditional indirect effects in Table 8.

**Figure 5:**
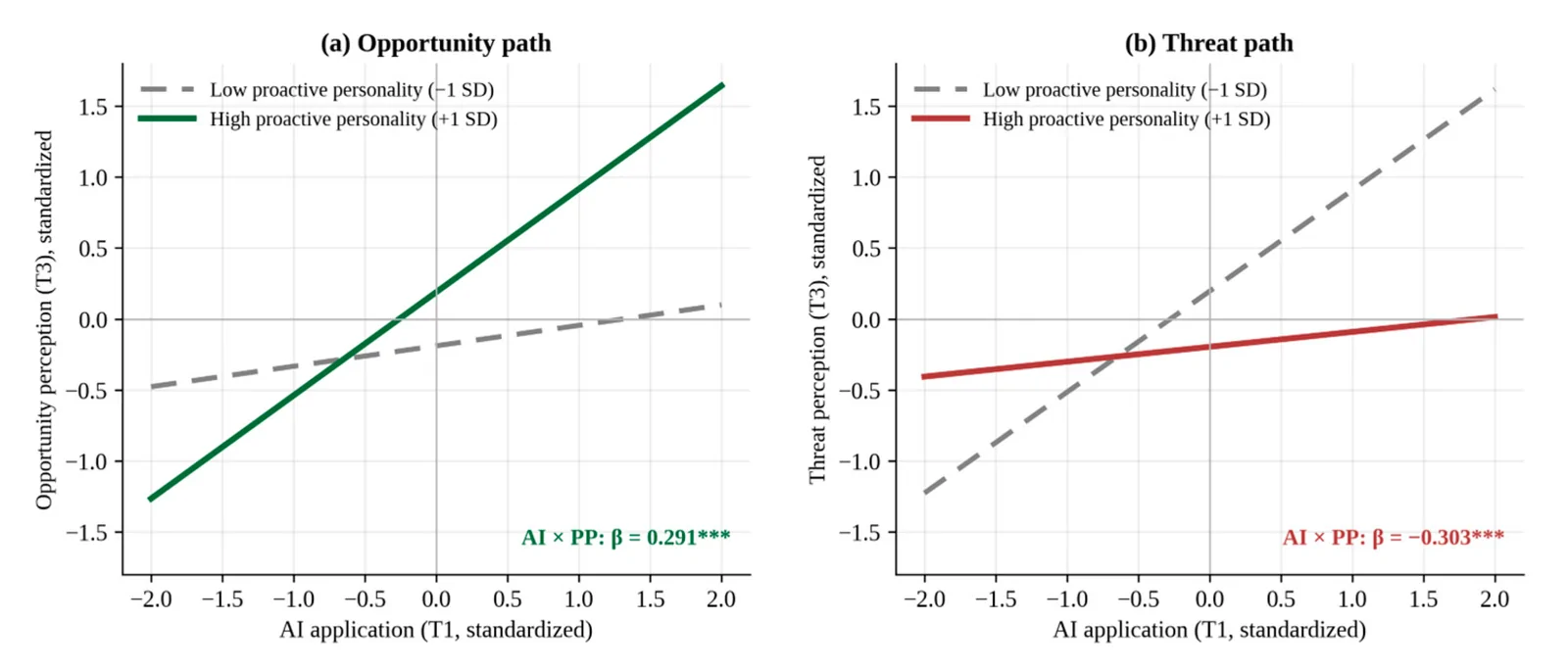
First-stage moderation of the AI application–appraisal relationships by proactive personality. *Note.* Simple slopes at ±1 *SD*. Lines show model-implied standardized scores based on Table 7 (Model 2); PP = proactive personality; β = standardized coefficient of the AI application × proactive personality interaction. All models control gender, age, education, tenure, hierarchical level, and baseline proactive learning behavior (T2). *** *p* < 0.001.

The AI Application × Proactive Personality interaction was significantly positive for opportunity perception (β = 0.291, *p* < 0.001, Δ*R*^2^ = 0.087) and significantly negative for threat perception (β = −0.303, *p* < 0.001, Δ*R*^2^ = 0.095). Simple slopes show that the association between AI application and opportunity perception rose from 0.144 at low proactive personality to 0.727 at high proactive personality, while the association with threat perception fell from 0.711 to 0.105 (Figure 5). These are tests of path moderation.

Turning to moderated mediation, the index was significant for both paths. For the opportunity path, the conditional indirect association rose from 0.065 at low proactive personality (95% CI [0.016, 0.112]) to 0.329 at high proactive personality (95% CI [0.252, 0.410]); IMM = 0.132 (95% CI [0.090, 0.180]), supporting H4a. For the threat path, the negative indirect association was attenuated from −0.213 (95% CI [−0.273, −0.155]) to −0.031 (95% CI [−0.062, −0.001]); IMM = 0.091 (95% CI [0.061, 0.124]), supporting H4b. Both direct associations hypothesized in H6a and H6b were also supported (β = 0.202 and β = −0.209, both *p* < 0.001).

We note that in the fully autoregressive specification the first-stage indices were not significant (0.017, 95% CI [−0.002, 0.040]; 0.001, 95% CI [−0.004, 0.009]). Consistent with Section 4.4, proactive personality is associated with the level of appraisal an employee forms, not with month-to-month revision of it.

### 4.7. Moderated Mediation at the Second Stage (Hypotheses 5a/5b and 6c)

We then added the Proactive Personality × Opportunity Perception and Proactive Personality × Threat Perception interactions to the equation predicting proactive learning (Table 9). Neither was significant (OPP × PP: β = 0.032, *p* = 0.282; THR × PP: β = 0.058, *p* = 0.071), and the incremental variance explained was negligible (Δ*R*^2^ = 0.003). The indices of moderated mediation were 0.013 (95% CI [−0.012, 0.045]) and 0.025 (95% CI [−0.002, 0.053]), both including zero (Table 10). H5a and H5b are not supported. Conditional indirect associations were stable across levels of proactive personality.

The direct association hypothesized in H6c was supported (β = 0.127, *p* < 0.001): proactive personality predicted proactive learning behavior over and above both appraisals and the baseline outcome. The null second-stage result does not indicate that proactive personality is behaviorally inert; it indicates specifically that proactive personality does not change the strength of the translation from a formed appraisal into learning. Employees high in proactive personality learn more, and read AI more favorably, but they do not convert a given appraisal into learning at a different rate.

Conditional indirect associations at both levels of proactive personality are reported in Table 10. A summary of all hypothesis tests is provided in Table 11.

### 4.8. Robustness Checks and Supplementary Analyses

**Figure 6:**
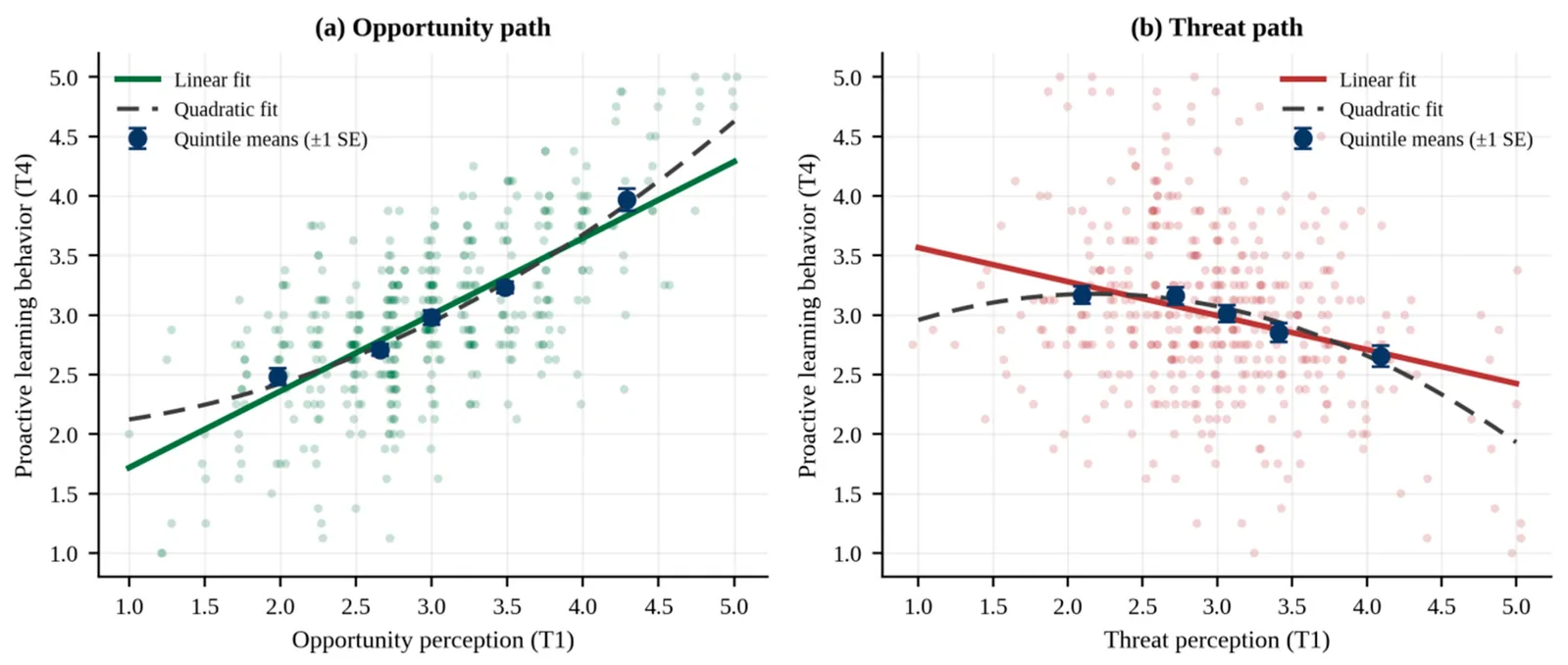
Functional form of the appraisal–learning relationships. *Note.* Panel (**a**) opportunity perception; panel (**b**) threat perception. Points are jittered observations; markers are quintile means with ±1 *SE*. Quadratic terms were non-significant for both mediators.

**Functional form.** We tested quadratic terms for both mediators alongside the controls, AI application, and the other mediator. The quadratic term was non-significant for threat perception (β = −0.032, *p* = 0.123, Δ*R*^2^ = 0.002) and for opportunity perception (β = −0.000, *p* = 0.983, Δ*R*^2^ < 0.001). Figure 6 overlays linear and quadratic fits with quintile group means for both mediators; group means decline monotonically for threat perception and rise monotonically for opportunity perception. The linear specifications in H2 and H3 are sustained for both appraisals, and the U-shaped and inverted-U alternatives can be set aside.

**Appraisal timescale.** Section 4.4 showed that AI application predicts the level but not the one-month change in threat perception. If the threat pathway operates on a longer horizon, then appraisals measured earlier should predict the outcome at least as well as more proximal ones. We therefore re-estimated the outcome equation using T1 appraisals (a three-month window), with the T4 outcome again adjusted for its T2 baseline. The threat coefficient was larger over the longer window (β = −0.363, *p* < 0.001) than over the one-month window (β = −0.319, *p* < 0.001), whereas the opportunity coefficient was smaller (β = 0.414 versus β = 0.476). The corresponding indirect associations were −0.164 (95% CI [−0.215, −0.116]) via threat perception and 0.192 (95% CI [0.142, 0.247]) via opportunity perception. The threat pathway is thus recoverable, and indeed stronger, when the appraisal–behavior window is extended. We report this as suggestive rather than conclusive: in this specification, the antecedent and the mediator are measured contemporaneously at T1, so it does not establish temporal precedence for the first stage. The proposal that the opportunity and threat pathways operate on different timescales is therefore offered as one plausible explanation of the observed asymmetry, not as a firm empirical conclusion. Competing explanations remain open, including the very high month-to-month stability of threat appraisal (*r* = 0.858), which leaves limited residual variance for AI application to predict, and the correspondingly limited power of the present design to detect genuine short-interval revision. Adjudicating among these accounts requires designs in which the appraisal window is varied deliberately rather than reconstructed post hoc.

**Industry heterogeneity.** To assess whether the three-item AI application measure behaves consistently across settings, we re-estimated the primary model within the six industry blocks with *n* ≥ 35 (Table 12). Both mediator–outcome paths retained their sign in every block, and the opportunity path was significant throughout. The threat–outcome path varied more in magnitude, from −0.035 in construction to −0.529 in public institutions, and the AI–opportunity path was weakest in services (0.169). We do not over-interpret differences among subsamples of 36 to 104 respondents, but the pattern is worth noting: the negative pathway appears strongest where employment is most formally structured. This is consistent with the view that our measure captures use intensity rather than technology subtype, and it reinforces the case for subtype-specific measurement.

**Alternative direction of influence.** A further question is whether proactive learning might shape subsequent appraisal rather than the reverse. We examined this in two ways. First, baseline proactive learning (T2) predicted T3 opportunity perception controlling for T1 opportunity perception (β = 0.061, *p* = 0.048) but not T3 threat perception controlling for T1 threat perception (β = −0.007, *p* = 0.789). Second, treating baseline proactive learning as a moderator rather than an antecedent, the AI Application × Baseline Learning interaction was significant for both opportunity perception (β = 0.191, *p* < 0.001) and threat perception (β = −0.119, *p* = 0.008). Employees who were already learning proactively converted AI application into opportunity appraisal more readily and into threat appraisal less readily. We report these results because they qualify our framing: the appraisal–learning relationship is unlikely to be purely unidirectional, and a reciprocal model is a legitimate alternative that our design cannot adjudicate.

## 5. Discussion

The findings provide a more nuanced account of how AI application relates to employees’ proactive learning behavior. Overall, AI application is positively associated with proactive learning, but this association operates through two opposing appraisal pathways: opportunity perception facilitates proactive learning, whereas threat perception inhibits it. The opportunity pathway is more robust across longitudinal specifications, while the threat pathway appears more sensitive to temporal specification. Proactive personality also plays a location-dependent role, shaping how employees appraise AI application rather than how they translate already-formed appraisals into learning behavior.

### 5.1. Theoretical Contributions

First, this study contributes to research on the relationship between AI application and proactive learning behavior. Prior studies on AI in organizations have mainly examined outcomes such as task performance, creativity, job insecurity, technology resistance, and employment displacement (Long et al., 2020; Sheng et al., 2022; Tu et al., 2023). Less attention has been paid to proactive learning behavior, even though employees’ self-initiated learning is central to whether AI implementation produces adaptive value. Because proactive learning is forward-looking and self-initiated, it cannot be assumed to arise automatically from technological change. Our findings show that AI application is positively associated with later proactive learning behavior, suggesting that AI use can serve as an important contextual condition that encourages employees to update skills and knowledge. This extends the AI–employee behavior literature by shifting attention from distal performance outcomes to the learning processes through which employees adapt to intelligent technologies.

Second, this study contributes by identifying the parallel mediating mechanisms through which AI application is linked to proactive learning behavior. Existing AI–employee research has increasingly recognized that AI may generate both opportunity and threat appraisals (Brougham & Haar, 2018; M. Li et al., 2026; H. Wang et al., 2026), but the psychological pathways through which these appraisals shape proactive learning remain underdeveloped. Drawing on the cognitive appraisal theory of stress (Lazarus & Folkman, 1984), we distinguish opportunity perception and threat perception as two separable primary appraisals. The results show that opportunity perception transmits a positive association between AI application and proactive learning, whereas threat perception transmits a negative association. This finding advances double-edged-sword research by showing that the positive and negative consequences of AI application do not simply reflect different technologies or different employee groups; rather, they can arise from distinct appraisals of the same technological context.

Third, this study contributes to cognitive appraisal theory by clarifying the coexistence and temporal asymmetry of opportunity and threat appraisals in the AI context. Cognitive appraisal theory suggests that individuals may evaluate the same event in multiple ways (Lazarus & Folkman, 1984), yet empirical evidence on whether positive and negative appraisals are separable and jointly held has remained limited. Our results indicate that opportunity and threat perceptions are statistically distinct and are jointly elevated in a meaningful subgroup of employees, which supports a parallel rather than strictly bipolar account. Because this evidence is distributional rather than within-person, we speak of parallel appraisals and leave strict simultaneity as an open question. At the same time, the two pathways are not equally stable across longitudinal specifications: the opportunity pathway is more robust, whereas the threat pathway is more sensitive to the timing and form of baseline adjustment. One plausible reading is that opportunity appraisal is more closely tied to current technological affordances, while threat appraisal reflects a slower evaluation of occupational risk. We present this timescale account as an interpretation to be tested rather than as a demonstrated result: the extended-window analysis measures the antecedent and the mediator contemporaneously and so does not establish temporal precedence at the first stage, and the attenuation of the threat pathway under full autoregression is equally compatible with high appraisal stability and limited power to detect change. In this way, the study refines cognitive appraisal theory by highlighting the parallel nature of primary appraisals and by raising, rather than settling, the question of whether they follow different temporal dynamics.

Fourth, this study contributes to the literature on proactive personality by specifying where this disposition matters in a moderated mediation process. Prior research has often treated proactive personality as a general enhancer of proactive behavior, implying that proactive employees are more likely to act on favorable appraisals or overcome unfavorable ones (Parker et al., 2006; Yan et al., 2025). Our results provide a more precise account. Proactive personality directly predicts opportunity perception, threat perception, and proactive learning behavior, but its moderating effect appears at the appraisal-formation stage rather than at the appraisal-to-behavior stage. This suggests that proactive personality functions primarily as a cognitive filter: employees high in proactive personality are more likely to interpret AI application as an opportunity and less likely to interpret it as a threat. The finding advances research on proactive personality by showing that its role in adaptation to AI lies less in strengthening behavioral responses to appraisals and more in shaping the appraisals themselves.

Relatedly, our findings invite a broader multilevel reading of where proactive responses to technology come from. Although the present design locates proactive personality at the individual level, employee innovation and other proactive responses are jointly shaped by higher-level contextual conditions and by individual psychological mechanisms. Multilevel evidence on the antecedents of employee innovation shows that the strategic orientation of senior leaders cascades downward through employees’ psychological states, and that the strength of this cascade depends on the external environment: X. Li et al. (2025) find that CEO entrepreneurial orientation is indirectly associated with employees’ innovative behavior through their felt responsibility for constructive change, with environmental turbulence strengthening that indirect association. Read alongside our results, this suggests that the cognitive filter we document is unlikely to operate in isolation. Whether employees convert a given level of AI application into opportunity rather than threat appraisal plausibly depends not only on dispositional proactivity but also on organizational signals about the technology, including how senior leaders frame AI adoption, whether the firm is entrepreneurially oriented, and how turbulent the technological environment is perceived to be. A fuller account would model such contextual conditions as cross-level moderators of the appraisal-formation stage, alongside dispositional moderators, so that contextual and dispositional routes to proactive responses can be compared rather than assumed to be independent. Our single-level design cannot test this, and we regard it as a priority for subsequent work.

Finally, this study offers a methodological contribution to research on AI-related employee adaptation. Many prior studies rely on cross-sectional or two-wave designs, which make it difficult to separate antecedents, mediators, and outcomes over time (Long et al., 2020; Sheng et al., 2022). By using a four-wave panel design, testing longitudinal measurement invariance, and comparing baseline-adjusted specifications, this study provides a more stringent examination of the proposed mediation process. Importantly, the results show that analytical choices concerning baseline adjustment can materially affect substantive conclusions, especially for the threat pathway. This highlights the value of treating longitudinal specification not merely as a robustness check but as part of theory testing in studies of AI, appraisal, and employee learning.

### 5.2. Practical Implications

Guide employees’ primary appraisals, not only their behavior. Because proactive personality exerts its influence at the appraisal stage, and because appraisals precede behavior, the upstream lever is how employees initially frame the arrival of AI. Positioning AI as a complementary partner rather than a replacement threat from the first interaction, through communication, pre-adoption training, and demonstration sessions emphasizing augmentation, is likely to matter most during onboarding, before threat appraisals consolidate. We offer this as a reasoned extrapolation: our design establishes association, not the efficacy of any intervention, and framing interventions of this kind have not been experimentally tested here.

Address opportunity and threat as separate targets. Our results indicate that threat perception is associated with lower proactive learning monotonically across its observed range, with no evidence of a motivating intermediate level. We stop short of the stronger claim that every unit reduction in threat is beneficial: we did not manipulate threat, we did not measure the costs of threat-reduction efforts, and monotonicity within an observed range does not warrant a prescription about all levels. What the evidence does support is that organizations should not rely on productive anxiety to motivate upskilling, and that reskilling programs, career transition support, and transparent job-scope clarification are complements to, not substitutes for, programs highlighting AI’s benefits.

Develop proactive orientation; do not merely select for it. Proactive personality predicts more favorable appraisals and more learning, but it does not accelerate the conversion of appraisal into learning, and employees low in proactive personality are precisely those most at risk of crystallizing threat appraisals. Selection is therefore a partial lever at best. The more promising and more equitable strategy combines contextual design (training ecosystems, psychological safety, transparent career pathways) with efforts to develop a more proactive orientation among incumbent employees, for instance through job crafting support, structured autonomy, and feedback practices that reward initiative. Evidence on human–AI collaboration and employee outcomes points in a similar direction (Wu et al., 2025a, 2025b).

### 5.3. Limitations and Future Directions

First, despite four-wave temporal separation, all measures are employee self-reports. Our common method checks are diagnostic only, and some inflation of observed associations cannot be excluded. Supervisor ratings of proactive learning, system logs of AI engagement, or learning-platform records would provide the triangulation this study lacks.

Second, our design cannot adjudicate the direction of influence between appraisal and proactive learning. Section 4.8 shows that baseline learning predicts subsequent opportunity appraisal and moderates the AI–appraisal relationship, so a reciprocal model is plausible. Cross-lagged panel models with more waves, or random-intercept specifications separating within- from between-person variance, would be better suited to this question.

Third, the threat pathway is supported when the outcome is baseline-adjusted but not when the mediator is additionally residualized, and it is stronger over a three-month than a one-month window. The one-month interval was not optimal for this pathway. Experience-sampling or latent growth designs with more than four waves, and ideally with intervals varying by construct, are needed to characterize the temporal structure of threat appraisal.

Fourth, the parallel-appraisal evidence is distributional rather than within-person. Establishing that two appraisals are simultaneously active within a moment of experience requires intensive longitudinal designs that we did not employ.

Fifth, our three-item AI application measure captures self-reported use intensity and cannot distinguish generative AI, robotic process automation, decision-support systems, or other subtypes; the industry-block variation reported in Section 4.8 suggests this distinction may matter. The sample is also drawn from a single cultural context. Subtype-specific measurement and cross-cultural replication are both needed.

Sixth, we did not assess secondary appraisal. Because coping-resource evaluation is theorized to condition whether threat appraisal produces withdrawal or problem-focused learning, including it would allow a fuller test of the cognitive appraisal model and might explain individual variation in the threat pathway.

Seventh, all constructs were modeled at the individual level, and respondents were not nested within identifiable organizations, so we could not examine the contextual conditions that plausibly shape appraisal formation. Since proactive and innovative employee responses are jointly determined by higher-level conditions and individual psychological mechanisms (X. Li et al., 2025), multilevel designs that combine organization-level variables such as leader entrepreneurial orientation, AI implementation climate, and perceived environmental turbulence with individual dispositions would situate the role of proactive personality within a broader perspective and allow cross-level moderation of the appraisal-formation stage to be tested directly.

## 6. Conclusions

Using a four-wave panel design among 420 employees in eight industries, we examined how AI application relates to proactive learning behavior through two parallel primary appraisals. Three findings stand out: First, with the outcome adjusted for its baseline, AI application was positively associated with proactive learning overall, and this net association combined a positive pathway through opportunity perception with a negative pathway through threat perception. The two pathways are not equally well evidenced: the opportunity pathway survived every specification we estimated, whereas the threat pathway did not survive full autoregressive adjustment and was recoverable only over a longer appraisal window. A difference in the timescales on which the two appraisals operate is a plausible explanation of this pattern, and one we prefer to the view that the threat pathway is spurious, but our design does not establish it; the asymmetry itself is real and is reported as such. Second, the two appraisals were statistically independent and jointly elevated in a substantial minority of employees, which is consistent with parallel appraisal and inconsistent with strict bipolarity, though this distributional evidence falls short of demonstrating within-person simultaneity. Third, proactive personality predicted both appraisals and proactive learning directly, and moderated the first stage rather than the second, functioning as a filter on how AI is read rather than as an amplifier of appraisals already formed. Taken together, these results extend the cognitive appraisal framework to the AI domain, raise, rather than settle, the question of a temporal dimension in its account of parallel primary appraisals, and show that testing moderation at a single assumed location can mischaracterize where individual differences actually operate.

## Figures and Tables

**Table 1:** Confirmatory Factor Analysis Results (*N* = 420).

Model	χ^2^	*df*	χ^2^/*df*	RMSEA	CFI	TLI	SRMR
Five-factor (hypothesized)	304.83	289	1.055	0.011	0.998	0.997	0.030
Four-factor (OPP + THR merged)	1428.93	293	4.877	0.096	0.821	0.802	0.185
Three-factor (AI merged)	1960.40	296	6.623	0.116	0.738	0.712	0.208
Two-factor	2649.09	298	8.890	0.137	0.630	0.596	0.234
One-factor	3274.86	299	10.953	0.154	0.531	0.491	0.190

*Note.* OPP = opportunity perception; THR = threat perception; AI = AI application. χ^2^ = chi-square statistic; *df* = degrees of freedom; RMSEA = root mean square error of approximation; CFI = comparative fit index; TLI = Tucker–Lewis index; SRMR = standardized root mean square residual. Each successive model merges an additional construct into a single factor.

**Table 2:** Composite Reliability, Average Variance Extracted, and Scale Reliability.

Construct	Loading Range	α	CR	AVE	√AVE
AI application (T1)	0.700–0.736	0.848	0.848	0.651	0.807
Opportunity perception (T3)	0.712–0.751	0.872	0.872	0.631	0.794
Threat perception (T3)	0.619–0.751	0.910	0.910	0.593	0.770
Proactive personality (T1)	0.648–0.723	0.841	0.841	0.569	0.754
Proactive learning behavior (T4)	0.660–0.735	0.927	0.927	0.615	0.784

*Note.* α = Cronbach’s alpha; CR = composite reliability; AVE = average variance extracted; √AVE = square root of AVE. CR ≥ 0.70 and AVE ≥ 0.50 are the recommended thresholds. Baseline scale reliabilities: opportunity perception T1 α = 0.859; threat perception T1 α = 0.913; proactive learning behavior T2 α = 0.926.

**Table 3:** Longitudinal Measurement Invariance Tests (*N* = 420).

Construct and Model	χ^2^	*df*	CFI	RMSEA	Δχ^2^	Δ*df*	ΔCFI	ΔRMSEA
*Opportunity perception (T1* vs. *T3)*								
Configural	13.25	13	1.000	0.007	—	—	—	—
Metric (equal loadings)	14.58	17	1.000	0.000	1.33	4	0.000	−0.007
Scalar (equal intercepts)	17.99	21	1.000	0.000	3.41	4	0.000	0.000
*Threat perception (T1* vs. *T3)*								
Configural	76.64	67	0.997	0.019	—	—	—	—
Metric	81.51	74	0.998	0.016	4.88	7	+0.001	−0.003
Scalar	91.20	81	0.997	0.017	9.69	7	−0.001	+0.001
*Proactive learning behavior (T2* vs. *T4)*								
Configural	112.18	93	0.996	0.022	—	—	—	—
Metric	114.72	101	0.997	0.018	2.54	8	+0.001	−0.004
Scalar	126.74	109	0.996	0.020	12.02	8	−0.001	+0.002

*Note.* CFI = comparative fit index; RMSEA = root mean square error of approximation; *df* = degrees of freedom; Δ = change relative to the preceding, less constrained model. Residual correlations were specified between same-item pairs across waves. All |ΔCFI| ≤ 0.001 and |ΔRMSEA| ≤ 0.007, meeting the criteria of Cheung and Rensvold (2002). Scalar invariance is supported for all three constructs.

**Table 4:** Descriptive Statistics and Intercorrelations (*N* = 420).

Variable	*M*	*SD*	1	2	3	4	5	6	7	8	9	10	11	12
1. Gender	1.53	0.50												
2. Age	1.95	0.64	−0.048											
3. Education	2.59	0.88	−0.004	0.071										
4. Organizational tenure	3.02	1.37	−0.044	−0.048	−0.008									
5. Hierarchical level	1.77	0.95	0.025	−0.057	−0.122 *	−0.068								
6. AI application (T1)	3.01	0.79	0.076	−0.029	−0.045	−0.067	−0.070							
7. Opportunity perception (T1)	3.00	0.76	0.046	−0.037	0.089 ^†^	−0.068	−0.001	0.460 ***						
8. Threat perception (T1)	2.98	0.70	0.077	−0.035	−0.045	−0.071	−0.056	0.463 ***	−0.043					
9. Proactive learning behavior (T2)	3.00	0.73	0.006	−0.029	0.039	−0.034	−0.047	0.192 ***	0.444 ***	−0.059				
10. Opportunity perception (T3)	3.02	0.76	0.062	−0.071	0.061	−0.060	−0.002	0.468 ***	0.823 ***	−0.022	0.415 ***			
11. Threat perception (T3)	2.99	0.72	0.095 ^†^	−0.043	−0.056	−0.033	−0.086 ^†^	0.425 ***	−0.024	0.858 ***	−0.056	0.007		
12. Proactive personality (T1)	2.99	0.73	0.043	−0.026	0.062	−0.062	0.028	0.005	0.315 ***	−0.219 ***	0.509 ***	0.328 ***	−0.223 ***	
13. Proactive learning behavior (T4)	3.00	0.72	0.082 ^†^	−0.001	0.041	−0.058	−0.010	0.330 ***	0.671 ***	−0.276 ***	0.533 ***	0.678 ***	−0.247 ***	0.459 ***

*Note. N* = 420. *M* = mean; *SD* = standard deviation. All correlations are reported in the body of the table. Gender was coded 1 = male, 2 = female. Cross-wave stability coefficients appear on the T1–T3 and T2–T4 diagonals (rows 10, 11, and 13). *** *p* < 0.001, * *p* < 0.05, ^†^ *p* < 0.10.

**Table 5:** Hierarchical Regression Results for the Mediation Tests Under Three Baseline-Adjustment Specifications (Standardized Coefficients; *N* = 420).

Predictor	OPP (T3)	THR (T3)	PLB (T4)	OPP (T3)	THR (T3)	PLB (T4)	OPP (T3)	THR (T3)	PLB (T4)
	Model A	Model A	Model A	Model B	Model B	Model B	Model C	Model C	Model C
Gender	0.021	−0.064	0.065 ^†^	0.025	0.062	0.068 *	0.019	0.030	0.067 *
Age	−0.062	−0.029	0.033	−0.052	−0.033	0.035	−0.039	−0.011	0.031
Education	0.091 *	−0.043	−0.011	0.076 ^†^	−0.037	−0.013	0.002	−0.021	−0.017
Organizational tenure	−0.027	−0.009	−0.019	−0.019	−0.012	−0.014	−0.003	0.027	−0.022
Hierarchical level	0.035	−0.066	−0.027	0.046	−0.071	−0.013	0.005	−0.039	−0.010
Proactive learning behavior (T2)	—	—	—	0.334 ***	−0.144 **	0.283 ***	0.061 *	−0.017	0.259 ***
Opportunity perception (T1)	—	—	—	—	—	—	0.722 ***	0.008	0.190 ***
Threat perception (T1)	—	—	—	—	—	—	−0.059 ^†^	0.839 ***	−0.258 ***
AI application (T1)	0.469 ***	0.412 ***	0.192 ***	0.406 ***	0.439 ***	0.182 ***	0.149 ***	0.031	0.203 ***
Opportunity perception (T3)	—	—	0.588 ***	—	—	0.476 ***	—	—	0.312 ***
Threat perception (T3)	—	—	−0.341 ***	—	—	−0.319 ***	—	—	−0.103 ^†^
*R* ^2^	0.233	0.191	0.553	0.339	0.211	0.619	0.695	0.741	0.647
*F*	20.85	16.21	63.57	30.24	15.70	73.90	103.70	130.51	67.96

*Note.* OPP = opportunity perception; THR = threat perception; PLB = proactive learning behavior. Model A is the lagged specification without baseline adjustment, reported for comparability only. Model B is the primary specification and adjusts for the baseline outcome (T2) in every equation. Model C is fully autoregressive and additionally adjusts for both baseline mediators (T1), so that the T3 mediators enter as residualized change. Every variable is labeled with its measurement wave. *** *p* < 0.001, ** *p* < 0.01, * *p* < 0.05, ^†^ *p* < 0.10.

**Table 6:** Bootstrap Estimates of the Indirect Associations (5000 Draws; *N* = 420).

Specification	Path	Estimate	Boot *SE*	95% CI *LL*	95% CI *UL*	Decision
Model A (no baseline adjustment)	AI → OPP (T3) → PLB (T4)	0.276	0.033	0.210	0.343	Significant
	AI → THR (T3) → PLB (T4)	−0.141	0.022	−0.185	−0.099	Significant
	Direct (*c*′)	0.192	0.041	0.113	0.272	Significant
	Total (*c*)	0.328	0.055	0.221	0.436	Significant
Model B (primary; baseline outcome adjusted)	AI → OPP (T3) → PLB (T4)	0.193	0.026	0.143	0.248	Significant
	AI → THR (T3) → PLB (T4)	−0.140	0.021	−0.183	−0.100	Significant
	Direct (*c*′)	0.182	0.039	0.106	0.258	Significant
	Total (*c*)	0.235	0.050	0.136	0.333	Significant
Model C (fully autoregressive)	AI → OPP (T3) → PLB (T4)	0.046	0.014	0.020	0.077	Significant
	AI → THR (T3) → PLB (T4)	−0.003	0.004	−0.013	0.004	Not significant
	Direct (*c*′)	0.203	0.040	0.124	0.284	Significant
	Total (*c*)	0.246	0.043	0.164	0.331	Significant
Extended window (T1 appraisals → T4)	AI → OPP (T1) → PLB (T4)	0.192	0.027	0.142	0.247	Significant
	AI → THR (T1) → PLB (T4)	−0.164	0.025	−0.215	−0.116	Significant

*Note.* Bias-corrected bootstrap confidence intervals. AI = AI application; OPP = opportunity perception; THR = threat perception; PLB = proactive learning behavior; Boot *SE* = bootstrap standard error; CI = confidence interval; *LL* = lower limit; *UL* = upper limit; *c*′ = direct association; *c* = total association. In Model B the decomposition is exact (0.193 − 0.140 + 0.182 = 0.235) because the baseline outcome enters every equation. In Model C the baseline outcome (T2) is retained in every equation alongside the baseline mediators (T1), so the indirect estimates refer to residualized change in the mediators rather than to their level. The extended-window rows use appraisals measured at T1 with the T4 outcome adjusted for its T2 baseline; the antecedent and mediator are contemporaneous in that specification, which is why it is reported as supplementary. CIs excluding zero indicate significance.

**Table 7:** Hierarchical Regression for First-Stage Moderation (Outcomes: Opportunity and Threat Perception at T3; *N* = 420).

Predictor	OPP Model 1	OPP Model 2	THR Model 1	THR Model 2
Gender	0.016	0.028	0.071 ^†^	0.059
Age	−0.049	−0.035	−0.036	−0.051
Education	0.067 ^†^	0.050	−0.028	−0.010
Organizational tenure	−0.010	−0.011	−0.022	−0.021
Hierarchical level	0.037	0.014	−0.061	−0.038
Proactive learning behavior (T2)	0.228 ***	0.184 ***	−0.034	0.012
AI application (T1)	0.425 ***	0.436 ***	0.419 ***	0.408 ***
Proactive personality (T1)	0.202 ***	0.189 ***	−0.209 ***	−0.196 ***
AI application × Proactive personality	—	0.291 ***	—	−0.303 ***
*R* ^2^	0.369	0.456	0.242	0.337
Δ*R*^2^	—	0.087	—	0.095
*F*	30.03	38.20	16.43	23.11

*Note.* OPP = opportunity perception; THR = threat perception. All predictors were standardized before interaction terms were formed. These are tests of path moderation; tests of moderated mediation appear in Table 8. *** *p* < 0.001, ^†^ *p* < 0.10.

**Table 8:** First-Stage Moderated Mediation: Conditional Indirect Associations (5000 Draws).

Path	Proactive Personality	Indirect Association	95% CI *LL*	95% CI *UL*	Decision
AI → OPP (T3) → PLB (T4)	Low (−1 *SD*)	0.065	0.016	0.112	Significant
	High (+1 *SD*)	0.329	0.252	0.410	Significant
	Index of moderated mediation	0.132	0.090	0.180	Significant (H4a supported)
AI → THR (T3) → PLB (T4)	Low (−1 *SD*)	−0.213	−0.273	−0.155	Significant
	High (+1 *SD*)	−0.031	−0.062	−0.001	Significant
	Index of moderated mediation	0.091	0.061	0.124	Significant (H4b supported)

*Note.* AI = AI application; OPP = opportunity perception; THR = threat perception; PLB = proactive learning behavior; *SD* = standard deviation; CI = confidence interval; *LL* = lower limit; *UL* = upper limit. Estimates are from the primary specification, which adjusts for the baseline outcome (T2). In the fully autoregressive specification the two indices were 0.017 (95% CI [−0.002, 0.040]) and 0.001 (95% CI [−0.004, 0.009]), neither significant. CIs excluding zero indicate significance.

**Table 9:** Hierarchical Regression for Second-Stage Moderation (Outcome: Proactive Learning Behavior at T4; *N* = 420).

Predictor	Model 1	Model 2	Model 3
Gender	0.077 ^†^	0.062 *	0.063 *
Age	0.016	0.036	0.036
Education	0.021	−0.016	−0.015
Organizational tenure	−0.034	−0.009	−0.010
Hierarchical level	0.014	−0.016	−0.017
Proactive learning behavior (T2)	0.532 ***	0.227 ***	0.228 ***
AI application (T1)	—	0.196 ***	0.203 ***
Opportunity perception (T3)	—	0.452 ***	0.436 ***
Threat perception (T3)	—	−0.299 ***	−0.280 ***
Proactive personality (T1)	—	0.127 ***	0.133 ***
Opportunity perception × Proactive personality	—	—	0.032
Threat perception × Proactive personality	—	—	0.058 ^†^
*R* ^2^	0.293	0.629	0.633
Δ*R*^2^	—	0.336	0.003
*F*	28.47	69.49	58.45

*Note.* All predictors were standardized before interaction terms were formed. Model 2 provides the tests of Hypotheses 6a–6c. *** *p* < 0.001, * *p* < 0.05, ^†^ *p* < 0.10.

**Table 10:** Second-Stage Moderated Mediation: Conditional Indirect Associations (5000 Draws).

Path	Proactive Personality	Indirect Association	95% CI *LL*	95% CI *UL*	Decision
AI → OPP (T3) → PLB (T4)	Low (−1 *SD*)	0.164	0.111	0.219	Significant
	High (+1 *SD*)	0.190	0.132	0.254	Significant
	Index of moderated mediation	0.013	−0.012	0.045	Not significant (H5a not supported)
AI → THR (T3) → PLB (T4)	Low (−1 *SD*)	−0.148	−0.198	−0.099	Significant
	High (+1 *SD*)	−0.098	−0.144	−0.053	Significant
	Index of moderated mediation	0.025	−0.002	0.053	Not significant (H5b not supported)

*Note.* AI = AI application; OPP = opportunity perception; THR = threat perception; PLB = proactive learning behavior; *SD* = standard deviation; CI = confidence interval; *LL* = lower limit; *UL* = upper limit. Estimates are from the primary specification, which adjusts for the baseline outcome (T2). The conditional indirect associations remain significant at both levels of proactive personality; what is absent is a difference between them. CIs including zero indicate non-significance.

**Table 11:** Summary of Hypothesis Tests.

Hypothesis	Content	Key Statistic (Primary Specification)	Decision
H1	AI application → PLB (positive)	*c* = 0.235, 95% CI [0.136, 0.333]	Supported
H2a	AI → OPP → PLB (positive indirect)	0.193, 95% CI [0.143, 0.248]	Supported
H2b	AI → THR → PLB (negative indirect)	−0.140, 95% CI [−0.183, −0.100]	Supported with qualification (not significant under full autoregressive adjustment)
H3a	OPP → PLB (positive)	β = 0.476, *p* < 0.001	Supported
H3b	THR → PLB (negative)	β = −0.319, *p* < 0.001	Supported
H4a	PP moderates AI → OPP (first stage)	IMM = 0.132, 95% CI [0.090, 0.180]	Supported
H4b	PP moderates AI → THR (first stage)	IMM = 0.091, 95% CI [0.061, 0.124]	Supported
H5a	PP moderates OPP → PLB (second stage)	IMM = 0.013, 95% CI [−0.012, 0.045]	Not supported
H5b	PP moderates THR → PLB (second stage)	IMM = 0.025, 95% CI [−0.002, 0.053]	Not supported
H6a	PP → OPP (positive direct)	β = 0.202, *p* < 0.001	Supported
H6b	PP → THR (negative direct)	β = −0.209, *p* < 0.001	Supported
H6c	PP → PLB (positive direct)	β = 0.127, *p* < 0.001	Supported

*Note.* IMM = index of moderated mediation; CI = 95% bias-corrected bootstrap interval. OPP = opportunity perception; THR = threat perception; PLB = proactive learning behavior; PP = proactive personality. All estimates come from the primary specification, which adjusts for the baseline outcome (T2).

**Table 12:** Focal Paths Within Industry Blocks (Primary Specification).

Industry Block	*n*	AI → OPP	AI → THR	OPP → PLB	THR → PLB
Services	104	0.169	0.611	0.366	−0.529
Manufacturing	98	0.503	0.351	0.428	−0.253
Public institutions	60	0.541	0.357	0.463	−0.128
High technology	43	0.341	0.108	0.643	−0.336
Construction	37	0.442	0.501	0.338	−0.035
Education	36	0.296	0.395	0.523	−0.158
Full sample	420	0.406	0.439	0.476	−0.319

*Note. n* = subsample size; AI = AI application; OPP = opportunity perception; THR = threat perception; PLB = proactive learning behavior. Standardized coefficients from the primary specification estimated separately within each industry block with *n* ≥ 35 (six of eight blocks; government and “other” were too small). Signs are consistent across all blocks. Subsample estimates are imprecise and are reported to assess stability rather than to support between-industry inference.

## Data Availability

The raw survey data are not publicly available in order to protect participant confidentiality. The analysis code, processed statistical output, and full measurement instrument are available from the corresponding author on reasonable request. All measurement items are reproduced in Appendix A.

## Appendix A. Measurement Items

All items were administered in Chinese following translation–back-translation (Brislin, 1980) and rated on a 5-point Likert scale (1 = *strongly disagree*, 5 = *strongly agree*). Items marked (R) were reverse-scored. This appendix is provided so that readers can evaluate the content validity of the appraisal measures directly.

**AI application (T1; 3 items; adapted from Tang et al., 2022, and W. Wang & Liu, 2025)**

1. Most of my work involves using artificial intelligence technology.
2. I spend a substantial share of my working time operating AI-based tools or systems.
3. Completing my core tasks depends on the AI technologies deployed in my work.

**Opportunity perception (T1 and T3; 4 items; adapted from Brougham & Haar, 2018, and M. Li et al., 2026)**

1. Using AI technology offers many benefits.
2. AI technology helps me acquire knowledge and skills I would not otherwise develop.
3. AI technology makes it easier for me to do my work well.
4. I see little that is valuable in the AI technology used in my work. (R)

**Threat perception (T1 and T3; 7 items; adapted from Brougham & Haar, 2018, and M. Li et al., 2026)**

1. I worry that AI will one day replace my job.
2. I am concerned that the skills I have built up will lose their value because of AI.
3. AI technology could make my professional expertise less important in this organization.
4. I feel uncertain about my career prospects because of the spread of AI at work.
5. I am confident that my role will remain necessary however far AI develops. (R)
6. The growth of AI in my field poses no risk to my position. (R)
7. I expect my professional standing to be unaffected by AI. (R)

**Proactive personality (T1; 4 items; Bateman & Crant, 1993)**

1. I am constantly on the lookout for new ways to improve my life and work.
2. Wherever I have been, I have been a powerful force for constructive change.
3. If I believe in an idea, no obstacle will prevent me from making it happen.
4. I excel at identifying opportunities.

**Proactive learning behavior (T2 and T4; 8 items; adapted from Zou et al., 2023)**

1. I continually work to improve my work-related skills.
2. I actively seek out new knowledge relevant to changes in my job.
3. I take the initiative to learn technologies before they are required of me.
4. I set my own learning goals for my professional development.
5. I look for opportunities to practice newly acquired skills at work.
6. I ask colleagues or supervisors for guidance when I encounter unfamiliar tasks.
7. I reflect on my work experience in order to identify what I still need to learn.
8. I invest my own time in developing capabilities that my future work may require.
